# Excited-State Engineering in Heteroleptic Ionic Iridium(III) Complexes

**DOI:** 10.1021/acs.accounts.0c00825

**Published:** 2021-02-22

**Authors:** Filippo Monti, Andrea Baschieri, Letizia Sambri, Nicola Armaroli

**Affiliations:** † Istituto per la Sintesi Organica e la Fotoreattività, Consiglio Nazionale delle Ricerche (ISOF-CNR), Via P. Gobetti 101, 40129 Bologna, Italy; ‡ Dipartimento di Chimica Industriale “Toso Montanari”, Università di Bologna, Viale Risorgimento 4, 40136 Bologna, Italy

## Abstract

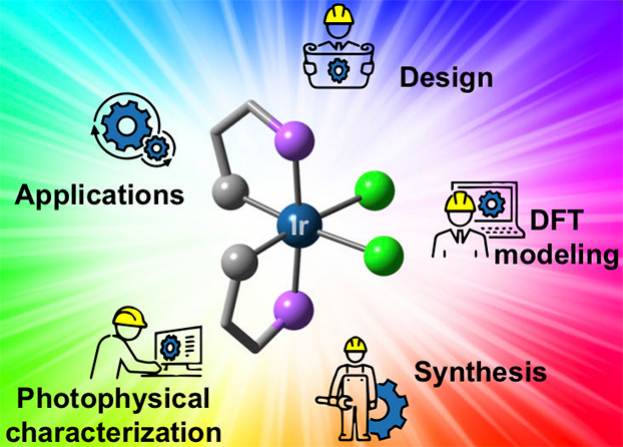

Iridium(III) complexes have assumed a prominent role in the areas of photochemistry and photophysics due to the peculiar properties of both the metal itself and the ligand environment that can be assembled around it. Ir(III) is larger, heavier, and bears a higher ionic charge than its analogue and widely used d^6^ ions such as Fe(II) and Ru(II). Accordingly, its complexes exhibit wider ligand-field d–d orbital splitting with electronic levels centered on the metal, typically nonemissive and photodissociative, not playing a relevant role in excited-state deactivations. In other words, iridium complexes are typically more stable and/or more emissive than Fe(II) and Ru(II) systems. Additionally, the particularly strong heavy-atom effect of iridium promotes singlet–triplet transitions, with characteristic absorption features in the UV–vis and relatively short excited-state lifetimes of emissive triplet levels. Ir(III) is also a platform for anchoring ligands of rather different sorts. Its versatile chemistry includes not only coordination with classic N^∧^N neutral ligands but also the binding of negatively charged chelators, typically having a cyclometalating C^∧^N anchor. The carbon–metal bond in these systems has some degree of covalent character, but this does not preclude a localized description of the excited states of the related complexes, which can be designated as metal-centered (MC), ligand-centered (LC), or charge transfer (CT), allowing a simplified description of electronic and photophysical properties. The possibility of binding different types of ligands and making heteroleptic complexes is a formidable tool for finely tuning the nature and energy of the lowest electronic excited state of cationic Ir(III) complexes by ligand design. Herein we give an account of our work on several families of iridium complexes typically equipped with two cyclometalating bidentate ligands (C^∧^N), in combination with mono or bidentate “ancillary” ligands with N^∧^N, C^∧^N, and C^∧^C motifs. We have explored new synthesis routes for both cyclometalating and ancillary ligands, obtaining primarily cationic complexes but also some neutral or even negatively charged systems. In the domain of the ancillary ligands, we have explored isocyanides, carbenes, mesoionic triazolylidenes, and bis-tetrazolic ligands. For the cyclometalating moiety, we have investigated carbene, mesoionic triazolylidene, and tetrazolic systems. Key results of our work include new strategies to modify both cyclometalating and ancillary ligands by relocating ionic charges, the determination of new factors affecting the stability of complexes, a demonstration of subtle structural effects that strongly modify the photophysical properties, new options to get blue-greenish emitters for optoelectronic devices, and a set of ligand modifications allowing the optimization of electrochemical and excited-state properties to obtain new promising Ir(III) complexes for photoredox catalysis. These results constitute a step forward in the preparation of custom iridium-based materials crafted by excited-state engineering, which is achieved through the concerted effort of computational and synthetic chemistry along with electrochemistry and photochemistry.

## Key References

- MontiF.; BaschieriA.; MatteucciE.; MazzantiA.; SambriL.; BarbieriA.; ArmaroliN.A chelating diisocyanide ligand for cyclometalated Ir(III) complexes with strong and tunable luminescence. Faraday Discuss.2015, 185, 233–24810.1039/C5FD00064E26400486.^1^*A novel chelating ancillary ligand imparting strong stability to isocyanide Iridium(III) complexes. By combining it with suitable cyclometalating chelators, it enables luminescence from deep blue to red*.
- MatteucciE.; BaschieriA.; MazzantiA.; SambriL.; ÁvilaJ.; PertegásA.; BolinkH. J.; MontiF.; LeoniE.; ArmaroliN.Anionic cyclometalated Iridium(III) complexes with a bis-tetrazolate ancillary ligand for light-emitting electrochemical cells. Inorg. Chem.2017, 56, 10584–1059510.1021/acs.inorgchem.7b0154428829579.^2^*An unprecedented dianionic ancillary ligand to build up a family of stable complexes with strong luminescence across the visible region (PLQYs of up to 0.83) and reversible electrochemical behavior, which can afford stable electroluminescent devices*.
- GualandiA.; MatteucciE.; MontiF.; BaschieriA.; ArmaroliN.; SambriL.; CozziP. G.Photoredox radical conjugate addition of dithiane-2-carboxylate promoted by an Iridium(III) phenyl-tetrazole complex: A formal radical methylation of Michael acceptors. Chem. Sci.2017, 8, 1613–162010.1039/C6SC03374A28451291PMC5364518.^3^*Our complexes at work. One of them is shown to be more effective and selective than the commercially available Ir-based standards for photoredox catalysis*.
- BaschieriA.; SambriL.; MazzantiA.; CarloneA.; MontiF.; ArmaroliN.Iridium(III) complexes with fluorinated phenyl-tetrazoles as cyclometalating ligands: Enhanced excited-state energy and blue emission. Inorg. Chem.2020, 59, 16238–1625010.1021/acs.inorgchem.0c0199533125213.^4^*One further step to the deep blue by modifying our tetrazolic cyclometalated ligands. The combination of relatively long lifetimes (ligand-centered states), high excited-state energy, and redox potentials opens the route to enhanced light-activated Ir-based catalysts for reductive quenching cycles*.

## 1 Introduction

Iridium was discovered in 1803, but for decades it had very limited practical applications. Its compounds exhibit high melting points and poor reactivity, which made them barely attractive for chemists, even less than compounds based on other precious transition elements.^5^ Iridium is possibly the rarest element on the earth’s crust, which did not increase its popularity,^6^ despite the potentially interesting physical and chemical properties of its compounds, which have been intensively exploited in photonics and optoelectronics only in the last two decades.^7^

Interest in the photochemistry of the so-called cyclometalated complexes started to increase at the beginning of the 1990s.^8^ These compounds entail carbon–metal bonds and are therefore borderline between classical Werner-type complexes and purely organometallic systems. Initially, the interest was mainly focused on Pd(II) and then it widened to Pt(II), Ru(II), Os(II), Rh(III), and Ir(III). The coordination chemistry of iridium is highly versatile because it can undergo both standard coordination with N-based chelators and cyclometalation. For instance, the 2,2′-bipyridine ligand in [Ir(bpy)_3_]^3+^ can bind the metal center with the classic N^∧^N or the less common C^∧^N anchor.^9^ Moreover, octahedral Ir(III) complexes can be mono-, bis-, and tris-cyclometalated, greatly widening the spectrum of structures potentially available, which can be either ionic or neutral.

In the area of the photochemistry and photophysics of coordination compounds, low-energy (UV–vis) electronic excitations are typically rationalized with a localized description of molecular orbitals, which can be, to a reasonable approximation, centered on the metal ion or the surrounding ligands. In this framework, electronic transitions can be categorized as metal-centered (MC), ligand-centered (LC), or charge-transfer (CT), with the latter occurring as metal-to-ligand, ligand-to-metal, or ligand-to-ligand charge transfer (MLCT, LMCT, or LLCT). Despite the fact that carbon–metal bonds have substantial covalent character and therefore a lower extent of localization, this description largely also holds for cyclometalated complexes.^8,10^ Similarly to standard coordination compounds, a judicious choice of the ligand environment, enables the design of cyclometalated complexes with tailored electronic properties once a synthesis route is established.^11^ In other words, the Ir(III) ion is a platform on which, by thoroughly designing the ligand environment, one can finely tune the nature and energy of the lowest electronic excited states and create complexes displaying specific UV–vis absorption and luminescence features, attractive electrochemical properties, and excited-state lifetimes spanning the microsecond to the second time scale.

Herein we give an account on our research journey of the excited-state engineering of cyclometalated Ir(III) complexes, presenting the exceptional versatility of these compounds as luminescent materials and photoredox catalysts.

## 2 Understanding Key Electronic Properties of Octahedral Cyclometalated Ir(III) Complexes

**Figure 1:**
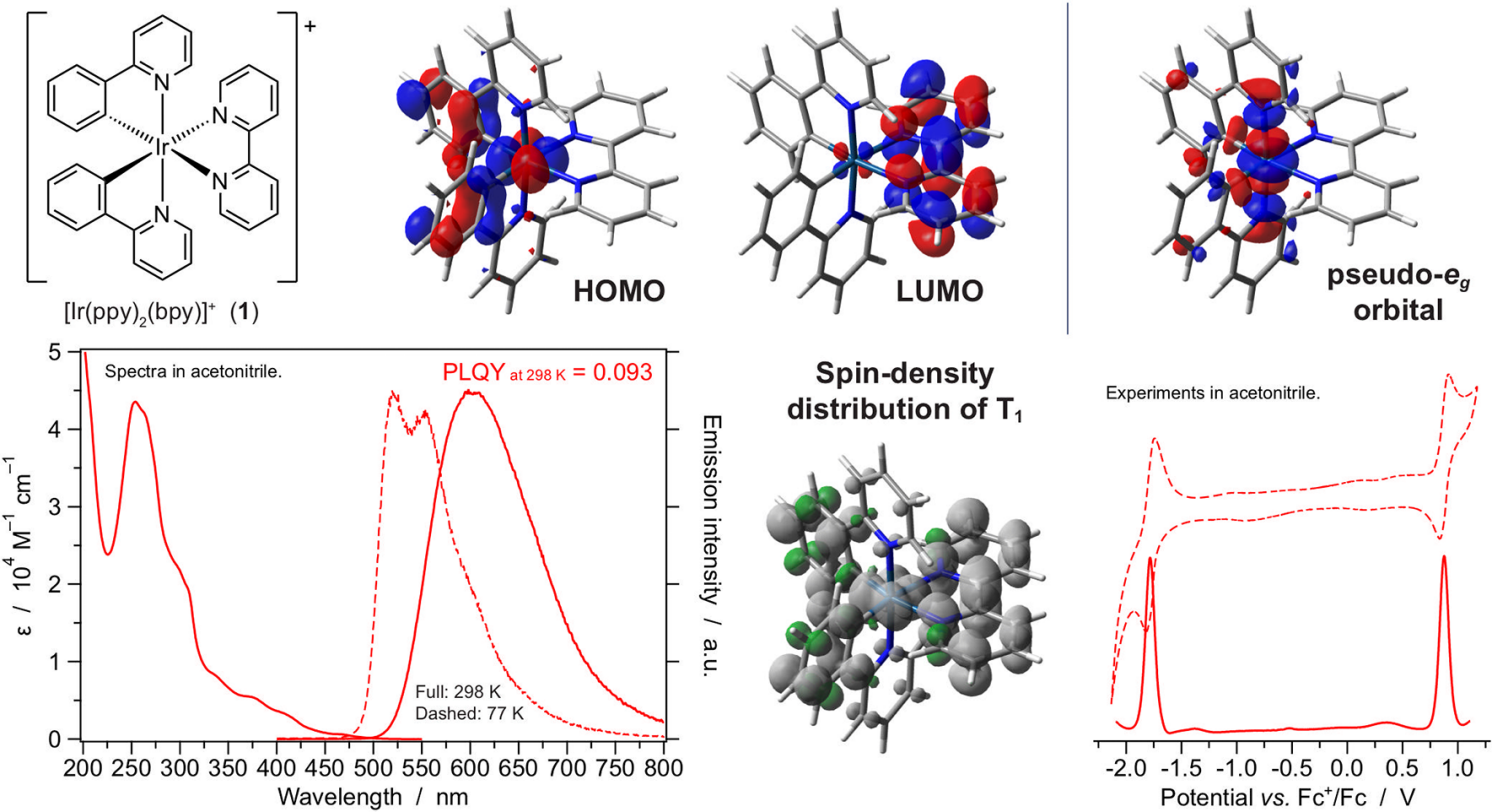
(Top) Chemical structure of [Ir(ppy)_2_(bpy)]^+^ (**1**), together with key molecular orbitals. (Bottom) Absorption and emission spectra in acetonitrile, spin-density distribution of the emitting triplet and electrochemical voltammograms of **1** (solid line, differential pulse voltammetry (DPV); dashed line, cyclic voltammetry (CV)).

By adopting the qualitative localized model mentioned above and exploiting the excellent predictive power of density functional theory (DFT) calculations, it is possible to gain a deeper understanding of the electronic properties of cyclometalated Ir(III) complexes, both in their ground state and lower-lying triplet excited states. Within this framework, [Ir(ppy)_2_(bpy)]^+^ (**1**, Figure 1) can be taken as a simple “archetypal” complex for cationic cyclometalated Ir(III) complexes.

As shown in Figure 1, [Ir(ppy)_2_(bpy)]^+^ can be used to illustrate some key general properties of cyclometalated Ir(III) complexes.

- The HOMO is localized on the metal but includes a significant contribution of the phenyl ring of the cyclometalated ligands, which reflects the partially covalent character of the iridium–carbon bonds; the LUMO is essentially localized on the π-conjugated (bpy) ancillary ligand. Accordingly, the lowest-energy emitting state is expected to display mixed ^3^MLCT/^3^LLCT character (often simply indicated as ^3^MLCT), as confirmed by more computationally expensive spin-unrestricted DFT optimizations carried out on the lowest triplet state of **1** (Figure 1).
- Ir(III) complexes undergo considerable splitting of the d orbitals—wider than d^6^ analogues based on Ru(II) and Fe(II)—because of (i) the higher ionic charge of the metal, (ii) the larger size of the d orbitals, and (iii) the intrinsically strong field exerted by cyclometalating ligands. As a consequence, the iridium pseudo-e_g_ antibonding orbitals pointing directly at the chelating ligands are raised at high energy, well above the π* orbitals of the ligands and with no absorption features attributable to MC electronic transitions in the UV–vis spectrum. The limited accessibility to MC states, which are known to be nonemissive or even undergo photodissociation, is a remarkable feature of Ir(III) complexes with respect to Ru(II) and Fe(II) d^6^ analogues, which are highly penalized for luminescence output or even stability due to deactivation from MC levels.
- The Ir(III) low-energy pseudo-t_2g_ orbitals are completely filled (low-spin d^6^ configuration), and the ground state is a singlet. The UV–vis absorption spectrum of the archetypal [Ir(ppy)_2_(bpy)]^+^ is dominated by distinct groups of bands corresponding to singlet excitations of a different nature (i.e., ^1^LC (centered on the two ligands, which span the UV region down to about 350 nm) and weaker ^1^MLCT/^1^LLCT at about 350–450 nm.
- Characteristic (very) weak absorption features are observed beyond 450 nm. These are related to the spin-forbidden transition directly populating triplet excited states (^3^MLCT, ^3^LLCT, and ^3^LC) thanks to the strong spin–orbit coupling induced by the heavy iridium ion.
- The luminescence band of [Ir(ppy)_2_(bpy)]^+^ centered at around 600 nm is attributed to the deactivation of the lowest ^3^MLCT excited state. It exhibits a lifetime of 0.3 μs in oxygen-free acetonitrile, which is a relatively short value due to the above-mentioned spin–orbit coupling which also favors triplet deactivation to the singlet ground state. It should be emphasized that lifetimes on the order of hundreds of nanoseconds are typical of ^3^MLCT states, while for emitting states having comparable emission intensity but more pronounced ^3^LC character, longer lifetimes are usually observed, reaching a few tens of microseconds.

The above points suggest that a rational modification of the structure and nature of the ligands, with respect to the “archetypal” [Ir(ppy)_2_(bpy)]^+^, may tune the key parameters that ultimately define the suitability of newly designed Ir(III) complexes for optoelectronic or photoredox applications. These include the emission color (depending on the band positioning and width across the visible spectrum), the luminescence intensity (quantified through the photoluminescence quantum yield, PLQY), the excited-state lifetime (τ), and the redox potentials (*E*_ox_, *E*_red_).

## 3 Preparative Strategies

Heteroleptic Ir(III) complexes typically entail two bidentate cyclometalating ligands (HC^∧^N), often identical, and an ancillary ligand (L or X^∧^Y) that saturates the coordination sphere of the iridium center. Our attention is mainly focused on octahedral complexes with the general formula [Ir(C^∧^N)_2_(X^∧^Y)]^*n*^, where *n* = +1, 0, −1.

**Figure 2:**
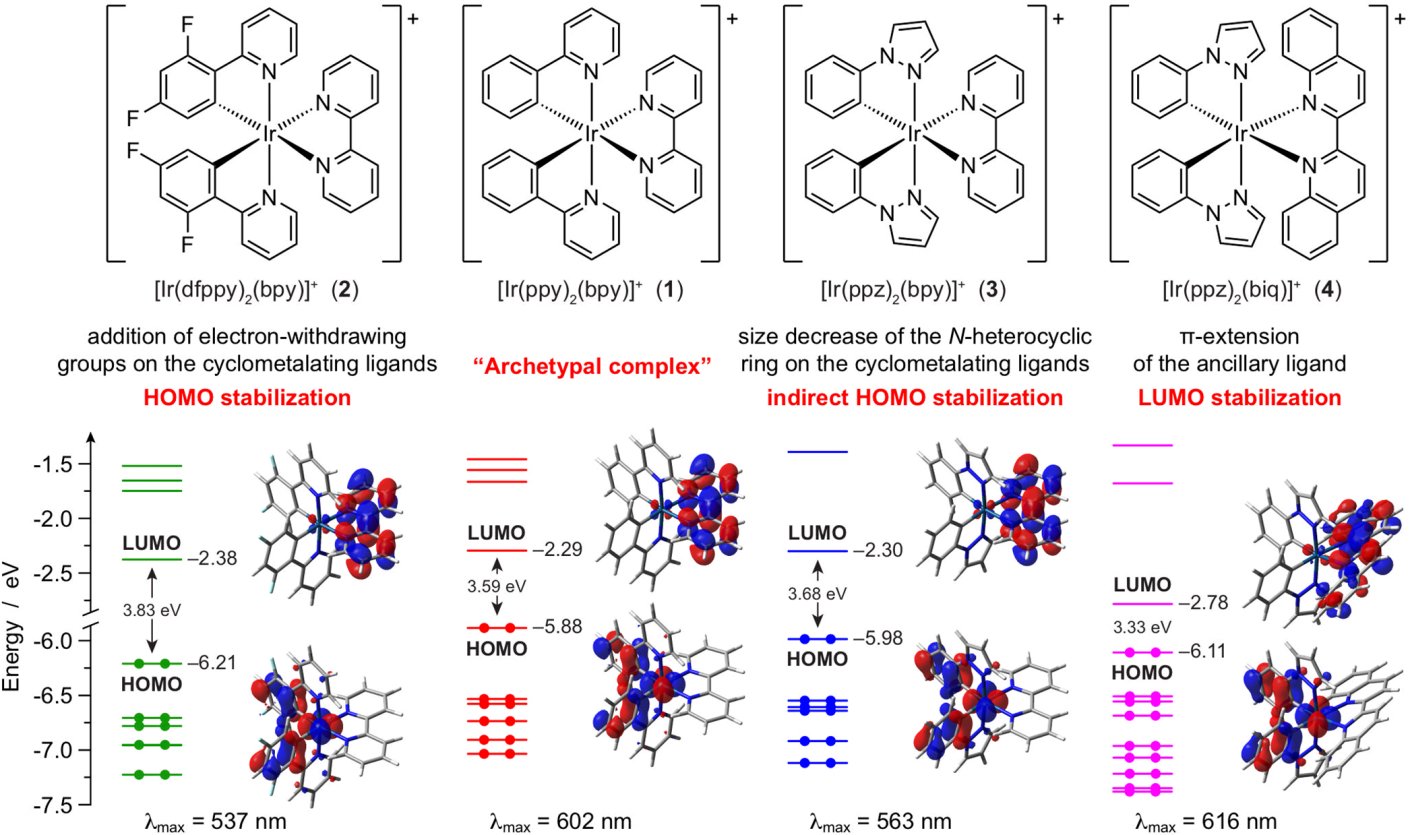
Chemical structures (top) and energy diagrams (bottom) of **1**–**4**. The effect of the ligand substituents on the frontier molecular orbitals of the related complexes is also displayed, together with the calculated HOMO–LUMO energy gap. The emission maximum in room-temperature acetonitrile solution is also reported, as an experimental determination of the energy of the emissive triplet state, to be correlated with the HOMO–LUMO gap.

Besides computational protocols that may anticipate the nature of key electronic excited states, efficient preparative methodologies are necessary to obtain the designed complexes. From a synthesis point of view, research is oriented along two main directions: (i) the preparation of cyclometalating ligands and the setting up of the conditions for the cyclometalation step and (ii) the synthesis of ancillary ligands and the optimization of the final reaction with the cyclometalated Ir(III) precursor. The main scope of designing new Ir(III) complexes by thorough ligand modification is tuning the HOMO–LUMO gap in order to tailor the energy of the lowest electronic excited states across the visible spectral region and obtain materials capable of emitting light all the way from the blue to the red. The most utilized ligand modifications affecting the HOMO and/or the LUMO energy of Ir(III) complexes are briefly illustrated in Figure 2.

### 3.1 Cyclometalation Strategies

**Scheme 1:**
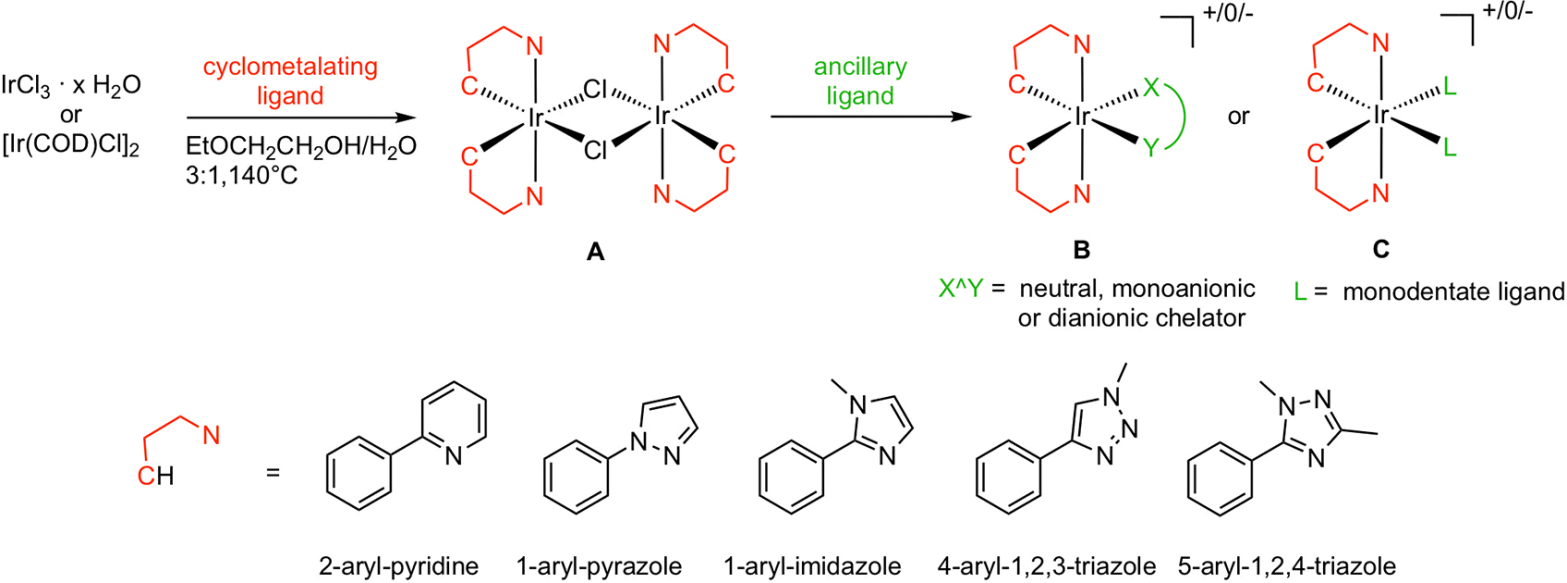
General Strategy for the Synthesis of Heteroleptic Cyclometalated Ir(III) Complexes

The most straightforward route to get Ir(III) precursors for luminescent complexes involves the direct cyclometalation of iridium(III) chloride hydrate (IrCl_3_·*x*H_2_O) with a specific HC^∧^N ligand at relatively high temperature to get chloro-bridged dimer [Ir(C^∧^N)_2_Cl]_2_ (**A** in Scheme 1).^12^

This reaction involves two steps: (i) coordination of the bidentate cyclometalating ligand to the metal center with a donor atom, usually nitrogen; (ii) removal of a proton on a suitable carbon atom to form a metal–carbon bond and generate a stable five-membered C–Ir–N ring (Scheme 1).

The cyclometalating ligands are formally monoanionic, and depending on the charge of the ancillary ligand, cationic, neutral, or anionic complexes can be prepared. This strategy works well with 2-aryl-pyridine,^13^ 1-aryl-pyrazole,^14^ 1-aryl-imidazole,^15^ 4-aryl-1,2,3-triazole,^16^ and 5-aryl-1,2,4-triazole^17^ derivatives (Scheme 1). Accordingly, in the last two decades hundreds of complexes have been obtained by reacting the related chloro-bridged dimers with a plethora of ancillary ligands.^10,12,18^

To increase the reactivity of the iridium salt and promote the cyclometalation reaction, the more reactive [Ir(COD)Cl]_2_ (COD = 1,5-cyclooctadiene) complex, where iridium has a +1 oxidation state, is sometimes used in combination with the HC^∧^N ligands to get the iridium dimer precursors.^19−21^ Contrary to IrCl_3_ that is cyclometalated via electrophilic aromatic substitution, [Ir(COD)Cl]_2_ usually undergoes a first oxidative addition due to the low oxidation state of the metal.^22^

### 3.2 Grafting the Ancillary Ligand

Once the procedures for obtaining cyclometalated Ir(III) precursors are established, attention is turned to the selection of the ancillary ligand. Bidentate chelators are typically preferred over monodentate ligands because the properties of the final complex are generally improved, particularly in terms of stability. Several commercially available compounds, such as bipyridines, arylpyridines, diketones, functionalized carboxylic acids, and monodentate isocyanides, have been successfully used to obtain neutral or charged luminescent Ir(III) complexes.^7^

The binding of the ancillary ligand is typically carried out under mild conditions, often at room temperature (Scheme 1). The Ir(III) dimer can be used as it is or can be treated with a Ag(I) salt (i.e., AgPF_6_, AgBF_4_, or AgOTf) to facilitate the removal of chlorine ions from the reaction environment, making the metal center more reactive and ready to bind. Ancillary ligands can be used directly (for example, N^∧^N diimine ligands) or can be treated with an Ag_2_O (for carbenes) or with a base to deprotonate NH or OH groups (for neutral complexes, typically used in OLED technology). As far as stereochemical considerations are concerned, iridium complexes used for photochemical studies are typically racemic forms of Λ and Δ enantiomers, carrying the nitrogen of the cyclometalating ligands in the trans position. When investigated with unpolarized light, such forms exhibit identical properties, though some differences have been evidenced in solid-state behavior.^23^

## 4 Ir(III) Complexes with Tailored Electronic Properties

### 4.1 Modifying the Ancillary Ligands

#### 4.1.1 Isocyanide Ancillary Ligands

The simplest way to tune the electronic properties of Ir(III) complexes is selecting an appropriate ancillary ligand for chloro-bridged dimer **A** (Scheme 1). We targeted this approach while looking for stable blue-emitting systems to be possibly utilized in optoelectronic devices, particularly light-emitting electrochemical cells (LECs).^10^ The basic idea is to “segregate” on the cyclometalated ligands and the iridium center the relevant low-energy excited states by selecting ancillary ligands which possess high-energy π* orbitals and cannot be readily involved in redox processes. The choice was addressed for neutral, strong-field, and nonchromophoric ancillary alkyl-^24,25^ and aryl-^14^ isocyanide ligands, affording several substituted derivatives.

**Figure 3:**
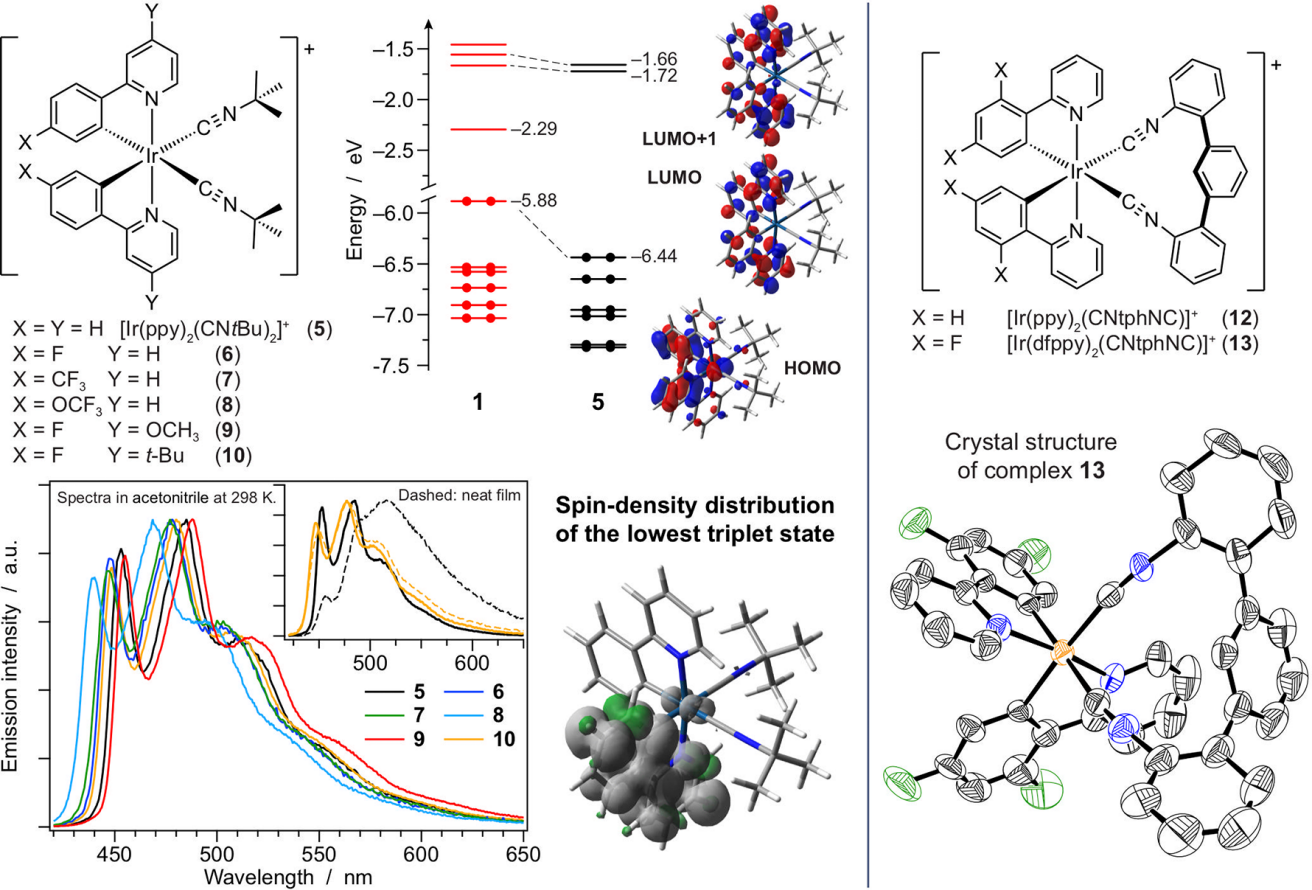
(Left) Chemical structures of **5**–**10**, together with their emission spectra. The energy diagram of **5** is also reported, compared to **1**, together with its frontier molecular orbitals and the spin-density distribution of the emitting triplet. (Right) Chemical formulas of **12** and **13** and X-ray structure of **13**, showing the 12-atom metallacycle.

These complexes display highly structured and intense ligand-centered emission bands (PLQY = 0.58 ± 0.09, Table S1) in acetonitrile, with the highest-energy feature in the 440–455 nm range (**5−9**, Figure 3) with relatively long lifetimes on the tens of microseconds time scale, as typical for ^3^LC states. Such a fine-tuning of the emission energy can be achieved by further stabilizing the HOMO or destabilizing the LUMO, depending on the presence of electron-withdrawing substituents (e.g., −F, −CF_3_, and −OCF_3_) on the Ir-phenyl fragment of the C^∧^N ligand (where the HOMO is located) and/or the addition of donating groups (e.g., −OCH_3_) on the pyridyl moiety of the same ligand (where the LUMO is found). The insertion of bulky *tert*-butyl groups (**10**) was found to be effective at preventing aggregation and yielding the first brightly blue-emitting isocyanide iridium complex in the solid state.^24^ Notably, the attempt to further push the emission at higher energy by destabilizing the LUMO with a smaller pyrazole ring instead of the standard pyridine on the C^∧^N ligand was successful, but at the expense of a dramatic drop in the PLQY (e.g., ∼0.001 for [Ir(ppz)_2_(CN*t*Bu)_2_]^+^ (**11**)).^14^ This is rationalized by the close proximity of thermally accessible ^3^MC states that deactivate nonradiatively.^26^ We further explored the use of isocyanide ancillary ligands by reporting the first example of a chelating diisocyanide, which forms complexes such as **12** and **13** (Figure 3) exhibiting an unusual 12-atom cycle containing iridium.^1^ These compounds display enhanced stability in solution compared to monodentate analogues and may afford luminescence from blue to orange upon extension of the π-conjugation from 2-phenylpyridine to 2-phenylquinoxaline.

**Figure 4:**
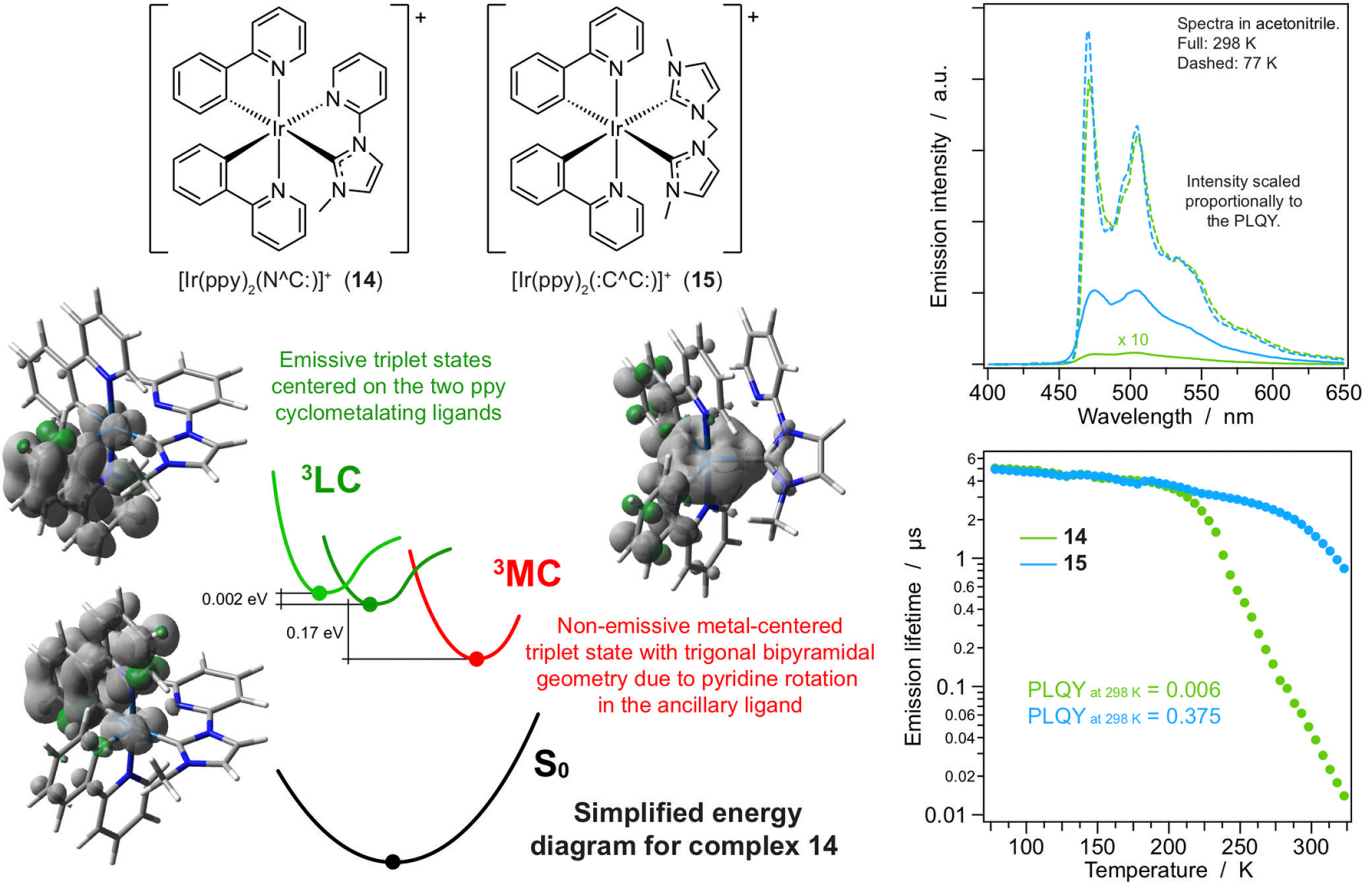
Chemical structures of **14** and **15**, together with their emission spectra (acetonitrile) and temperature-dependent lifetimes (butyronitrile). The complex interplay between emitting and nonradiative ^3^MC states is depicted for **14**.

**Figure 5:**
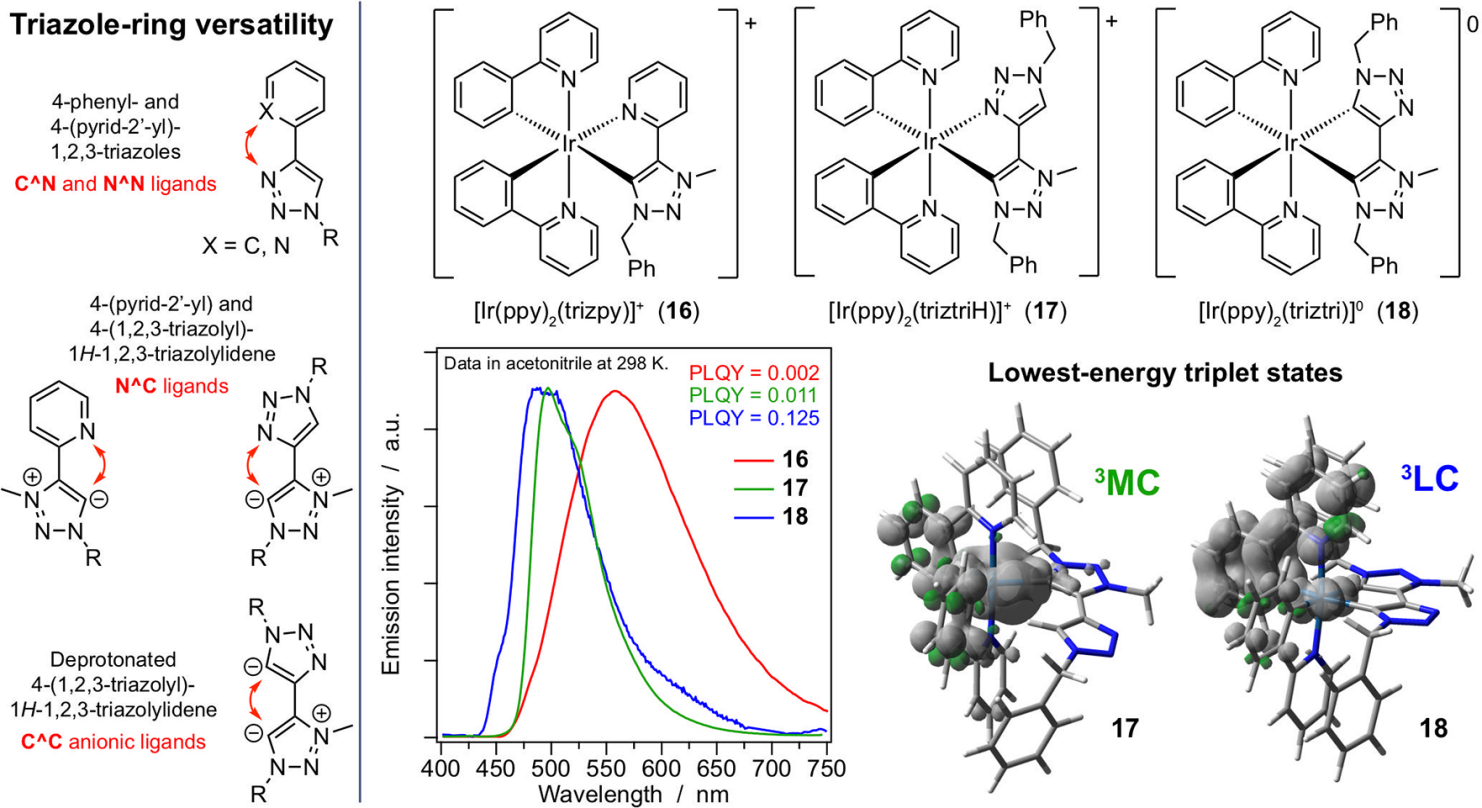
(Left) Different chelating modes of triazole-based ligands. (Right) Chemical structures of **16**–**18**, together with their emission spectra and PLQYs in acetonitrile. The different nature of the lowest triplet state is highlighted for **17** and **18**.

**Figure 6:**
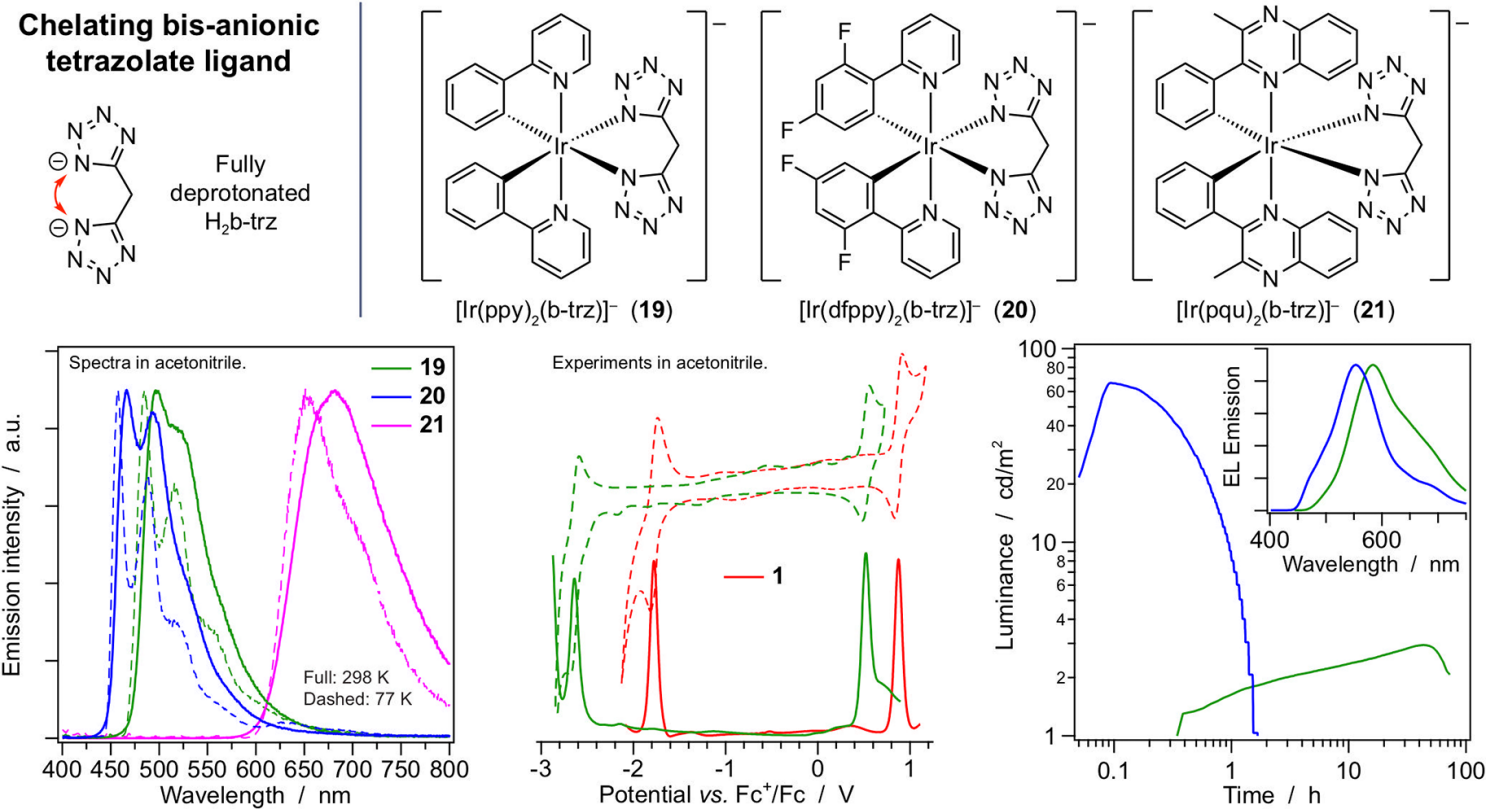
(Top) Chemical structure of the ancillary ligand and of related anionic iridium(III) complexes **19**–**21**. (Bottom) Emission spectra of **19**–**21** in acetonitrile; electrochemical voltammograms (DPV and CV) of **19** in acetonitrile, compared to **1**; luminance and electroluminescence spectra of LECs based on **19** and **20**.

**Figure 7:**
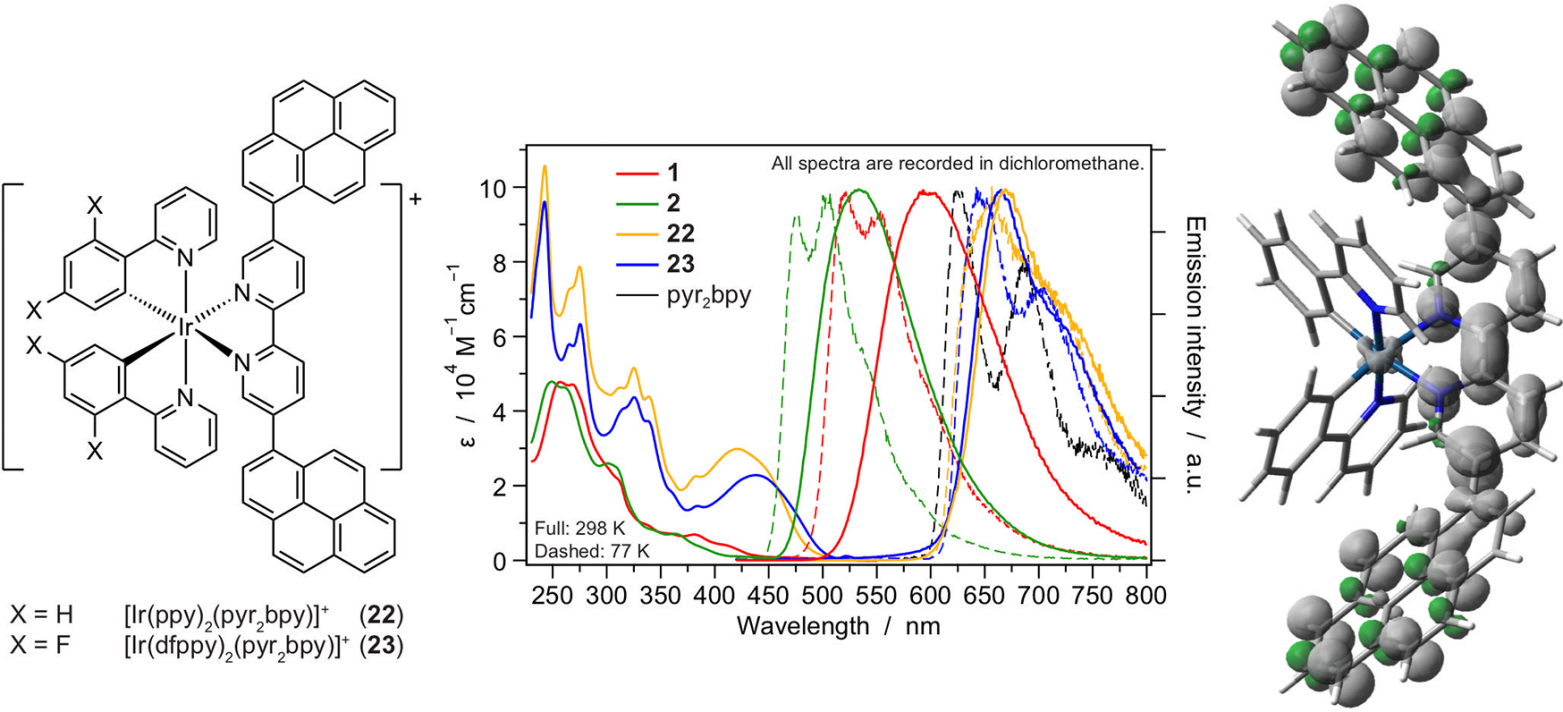
Chemical structures of **22** and **23**, together with their absorption and emission spectra in CH_2_Cl_2_. The spin-density distribution of the emitting state of **22** is also reported.

**Figure 8:**
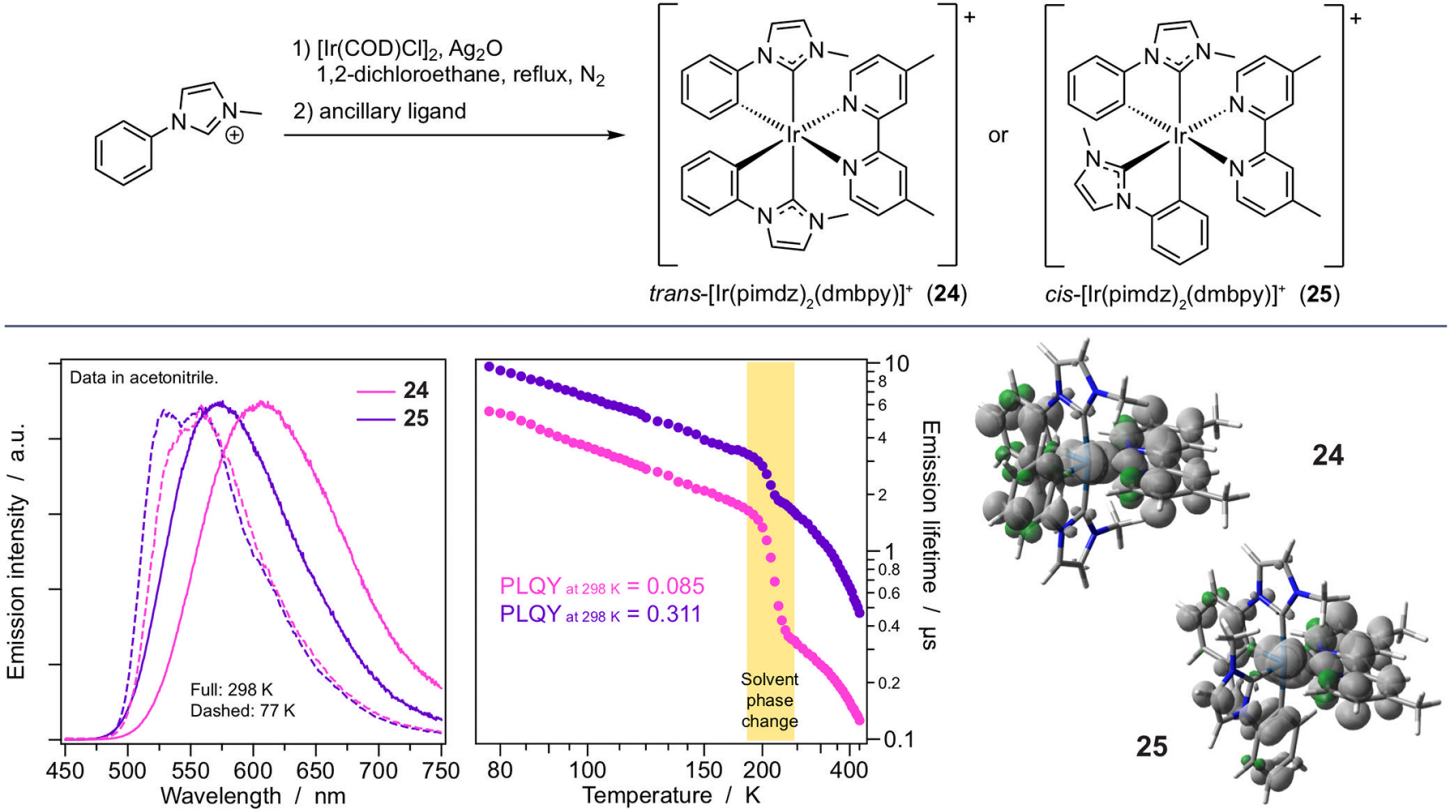
(Top) Synthesis strategy for the preparation of **24** and **25** using the imidazole-carbene C^∧^C: ligand. (Bottom) Emission spectra in acetonitrile, temperature-dependent lifetimes in propylene glycol, and spin-density distribution of the emitting states of **24** and **25**.

**Figure 9:**
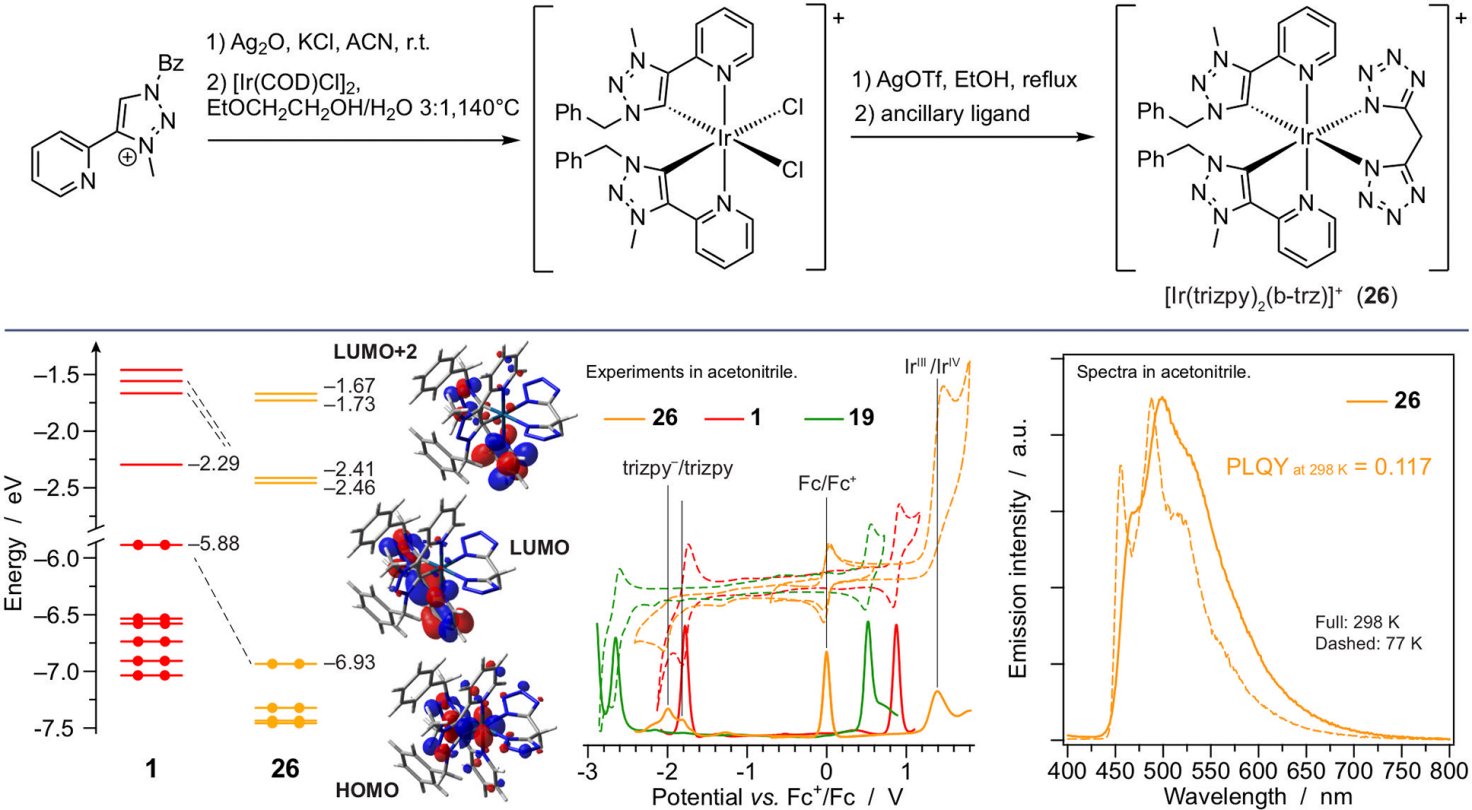
(Top) Synthesis strategy for the preparation of **26** using the mesoionic triazolylidene :C^∧^N ligand. (Bottom) Energy diagram, electrochemical voltammograms, and emission spectra of **26** in acetonitrile, with some comparisons with reference complexes.

#### 4.1.2 Carbene Ancillary Ligands

The use of pyridine-carbene (N^∧^C:) ancillary ligands was found to be an interesting alternative to standard chelating systems, such as 2,2′-bypiridine and 1,10-phenanthroline.^27,28^ In particular, similar to the above-described isocyanides, carbene-based imidazolylidene ancillary ligands can afford ligand-centered blue emission in cationic Ir(III) complexes by confining the HOMO and the LUMO on the cyclometalating moieties.^27,29^ However, these compounds may exhibit very poor PLQYs in room-temperature acetonitrile solution (∼0.01).^27^ By investigating in parallel imidazolylidene complexes bearing pyridine-carbene (N^∧^C:) and bis-carbene (:C^∧^C:) ligands (such as **14** and **15**, Figure 4), it was possible to rationalize this behavior and establish a route to strongly emitting systems with carbene ancillary ligands.^30^ The combination of DFT calculations and temperature-dependent spectroscopic studies showed that the lowest emissive ^3^LC state of **14** deactivates to a dark ^3^MC state by the decoordination of the pyridine ring in the N^∧^C: ligand (Figure 4). Such a level is higher-lying and hence not readily accessible in the bis-carbene analogue **15**. Consequently, **14** and **15** exhibit fully superimposable blue emission bands, but their PLQYs are, respectively, 0.006 and 0.375 in acetonitrile at 298 K. No significant differences in their photophysical behavior are found at low temperature or in a doped PMMA matrix, where the lack of thermal energy or conformational freedom prevents the population of the nonemissive ^3^MC state.

#### 4.1.3 Mesoionic Carbene Ancillary Ligands

Versatile triazole-based systems have been used to synthesize mesoionic carbenes, leading to complexes **16** and **17** (Figure 5). When an acidic proton is removed from the ring linked to the carbene moiety, the related monoanionic ancillary ligand can afford a neutral complex such as **18**.^31^ 1,2,3-Triazolylidene complexes behave similarly to the above-discussed imidazolylidene derivatives. **16** and **17** display low PLQY in acetonitrile (around 0.01), whereas **18**, obtained by switching the chelating mode of the ancillary ligand from N^∧^C: to a carbene-carbanion (:C^∧^C^–^), showed a 60-times higher PLQY. DFT calculations show that the luminescence of cationic **16** and **17** is quenched by the presence of low-lying ^3^MC states, leading to a reversible detachment of the neutral ancillary ligands from the iridium coordination sphere. This nonradiative deactivation pathway is absent in neutral complex **18**.^31^

#### 4.1.4 Dianionic Ancillary Ligand for Negatively Charged Complexes

A bis-tetrazole ancillary ligand (H_2_b-trz = di(1*H*-tetrazol-5-yl)methane, Figure 6) has been used, in combination with standard cyclometalating chelators, to afford an uncommon family of very stable negatively charged Ir(III) complexes.^2^ By changing the cyclometalating ligands along the series, **19**–**21** exhibit strong emission in the green, blue, and red spectral region (PLQYs of up to 0.83 in acetonitrile, Table S1). Compared to standard cationic complexes, this series is characterized by more negative redox potentials due to electrostatic effects. Green-emitting **19** exhibits very stable electroluminescence and was successfully utilized in a rare example of a light-emitting electrochemical cell (LEC) with an anionic active material.^2^ The device can stay at the maximum luminance for over 40 h, showing remarkable stability.

#### 4.1.5 Ancillary Ligand with Appended Chromophores

By grafting a suitable peripheral appendage, it is possible to avoid any direct role of the iridium center in the lowest triplet state of the related complexes. This was accomplished with **22**–**23**, where the emission stems from a charge-transfer state involving the ancillary ligand and the appended pyrene unit (Figure 7).^32^ This level acts as sink for the upper-lying excited states, including the typically emissive ^3^MLCT one that is commonly observable in the absence of pyrene. The PLQYs of **22**–**23** in CH_2_Cl_2_ are poor (<0.01), but this approach widens the possibilities for excited-state engineering in iridium complexes.^33,34^

### 4.2 Modifying the Cyclometalating Ligands

#### 4.2.1 Carbene Cyclometalating Ligands

Subtle structure-related effects on the excited states of Ir(III) complexes were evidenced by using phenyl-imidazoles as cyclometalating ligands in combination with a series of bipyridine or phenanthroline-type ancillary chelators.^35^ These carbene-type (C^∧^C:) complexes are characterized by the relatively strong yellowish emission of MLCT/LLCT nature in acetonitrile, with the ancillary ligand hosting the LUMO and serving as an electron acceptor. Luminescence and excited-state features are virtually the same regardless of the ancillary ligand, and only a minimal emission red shift is observed relative to the archetypal [Ir(ppy)_2_(bpy)]^+^. This family of compounds typically exhibits a *trans* configuration, with the imidazole rings in the apical positions of the octahedron (e.g., **24** in Figure 8). In one case, it was also possible to isolate the cis isomer (**25**, Figure 8), which shows peculiar excited-state behavior, with a blue-shifted emission band in room-temperature acetonitrile solution with respect to its *trans* analogue.^35^ The cis isomer is also characterized by a much higher PLQY (0.31 vs. 0.09) and a longer lifetime (1291 vs. 278 ns, Table S1) as a consequence of the 6-times-slower nonradiative deactivation pathway. Detailed temperature-dependent studies in propylene glycol evidenced that the different behavior of the two isomers at room temperature is attributable to solvation effects (i.e., a different ability of the dielectric medium to follow electronic and conformational changes of the excited-state while the complex relaxes to the T_1_ minimum-energy geometry).^35^ In a frozen matrix, such effects are eliminated and the *cis* and *trans* isomers have virtually identical photophysical properties.

#### 4.2.2 Neutral Cyclometalating Ligands

The synthesis strategy based on the [Ir(COD)Cl]_2_ reactant to get the cyclometalated solvato intermediate (Scheme 1) was implemented in the synthesis of Ir(III) complexes with a mesoionic carbene as a neutral bidentate ligand. In particular, we used 4-pyridyl-1,2,3-triazolylidene derivatives to obtain [Ir(trizpy)_2_Cl_2_]^+^ as a precursor (trizpy = 1-benzyl-3-methyl-4-(pyridin-2-yl)-1*H*-1,2,3-triazolylidene) and then related complex [Ir(trizpy)_2_(b-trz)]^+^ (**26**) through a simple synthesis procedure (Figure 9).^36^ The dianionic bis-tetrazole ancillary ligand (b-trz) allows us to retain the standard monocationic character of the complex. In **26**, the negative charge is moved to the ancillary ligand, and this represents a new concept in the area of cationic Ir(III) complexes. **26** exhibits a moderately intense ^3^LC emission band in the blue region in acetonitrile (λ_max_ = 499 nm; PLQY = 0.12), centered on the mesoionic chelator. A noteworthy feature is the high first oxidation potential, compared to complexes with standard negatively charged cyclometalating ligands, which reflects the neutral character of the mesoionic ligand, on which the HOMO is extensively located. Moreover, if compared to anionic counterpart **19** having the same ancillary ligand but standard 2-phenylpyridine cyclometalating units, **26** has a similar HOMO–LUMO gap but is shifted to more positive potentials due to its overall positive charge.

#### 4.2.3 Tetrazolic Cyclometalating Ligands

**Figure 10:**
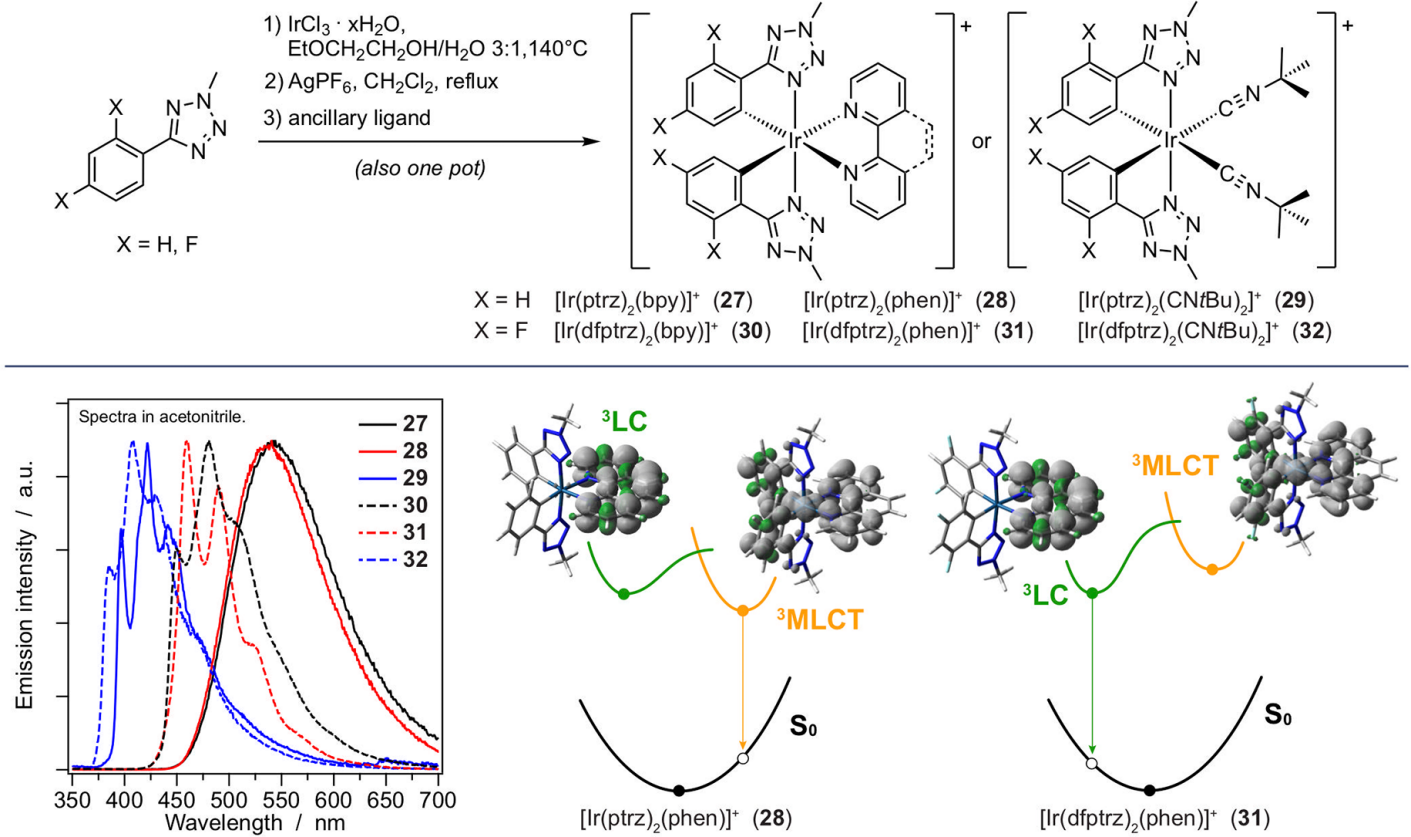
(Top) Synthesis strategy for the preparation of **27**–**32** using high-field phenyl-tetrazole ligands. (Bottom) Emission spectra of **27**–**32** in acetonitrile. The switching between ^3^LC and ^3^MLCT states is sketched for phenanthroline-based analogues **28** and **31** due to the effect of the fluorine substituents.

To achieve a blue-shifted emission in Ir(III) complexes, we tested high-field phenyl-tetrazoles as cyclometalating ligands (**27**–**29**, Figure 10).^37^ Furthermore, we also combined such ring-size reduction in the C^∧^N ligands with the further addition of electron-withdrawing groups (i.e., fluorine), as in **30**–**32**.^4^ Notably, all of the previously reported methodologies employing *N*-substituted phenyl-tetrazoles yielded an undefined iridium salt. Therefore, we developed a new silver-assisted reaction that makes the iridium core more reactive and generates cyclometalated solvato intermediate [Ir(ptrz)_2_(CH_3_CN)_2_]^+^ that is able to react with various ancillary ligands such as bipyridine and phenanthroline (**27** and **28**).^37^ These compounds exhibit a very strong and unstructured MLCT/LLCT emission in acetonitrile (PLQY > 0.55, Table S1), which was the highest-energy luminescence detected in cationic Ir(III) complexes without electron-withdrawing groups on the cyclometalating ligands (λ_max_ ≈ 540 nm, Figure 10).^37^ Eventually, the emission was further pushed to the blue by attaching fluorine substituents (λ_max_ ≈ 450 nm in **30** and **31**), but the nature of the excited states is radically different, with a strong and structured emission centered on the ancillary ligand and lifetimes substantially elongated (up to 10 times) with respect to those of nonfluorinated **27** and **28** due to the strong LC character of the excited states. The synthesis strategy for such fluorinated series is different from that of the unsubstituted analogues because it implies a one-pot procedure with the ancillary ligand added directly to the cyclometalated solvato intermediate without isolating it.^4^ By replacing the N^∧^N ancillary chelators with two monodentate isocyanide ligands (**29** and **32**), the emission of the related complexes is further pushed to the deep-blue region because the excited state is confined to the phenyl-tetrazole cyclometalating ligands. However, the room-temperature PLQY in acetonitrile is extremely weak (<0.01) but very strong at 77 K.^4,37^

## 5 Photoredox Catalysis

In recent years, Ir(III) cyclometalated complexes have also been examined to serve as light-stimulated catalysts for electron-transfer reactions,^38^ a capability that requires thorough excited-state engineering. The use of transition-metal complexes as visible-light photoredox catalysts for small-molecule activation or for the synthesis of organic building blocks has rapidly grown since the pioneering research by Yoon’s^39^ and MacMillan’s groups.^40^ Photoredox catalysts promote the conversion of light to chemical energy by readily generating radicals,^41^ which may act as reductants or oxidants depending on the reaction partner.^42^ In this way, in contrast to traditional redox reactions (e.g., electrochemistry), they prompt a redox-neutral reaction environment that is quite unique for organic chemistry. Light-mediated catalysis has found widespread applications in water splitting^43,44^ and carbon dioxide reduction^45^ as well as in one-electron radical processes for C–C bond formation.^42^ Notably, it enabled a variety of unconventional bond constructions, which were not attainable by conventional organic chemistry protocols.^46^ The mechanism of an outer-sphere reaction does not require a vacant coordination site on the catalyst because the substrate does not bind to the metal. The catalyst instead participates in single-electron-transfer (SET) processes with the organic substrates, providing facile access to open-shell reactive species.

**Figure 11:**
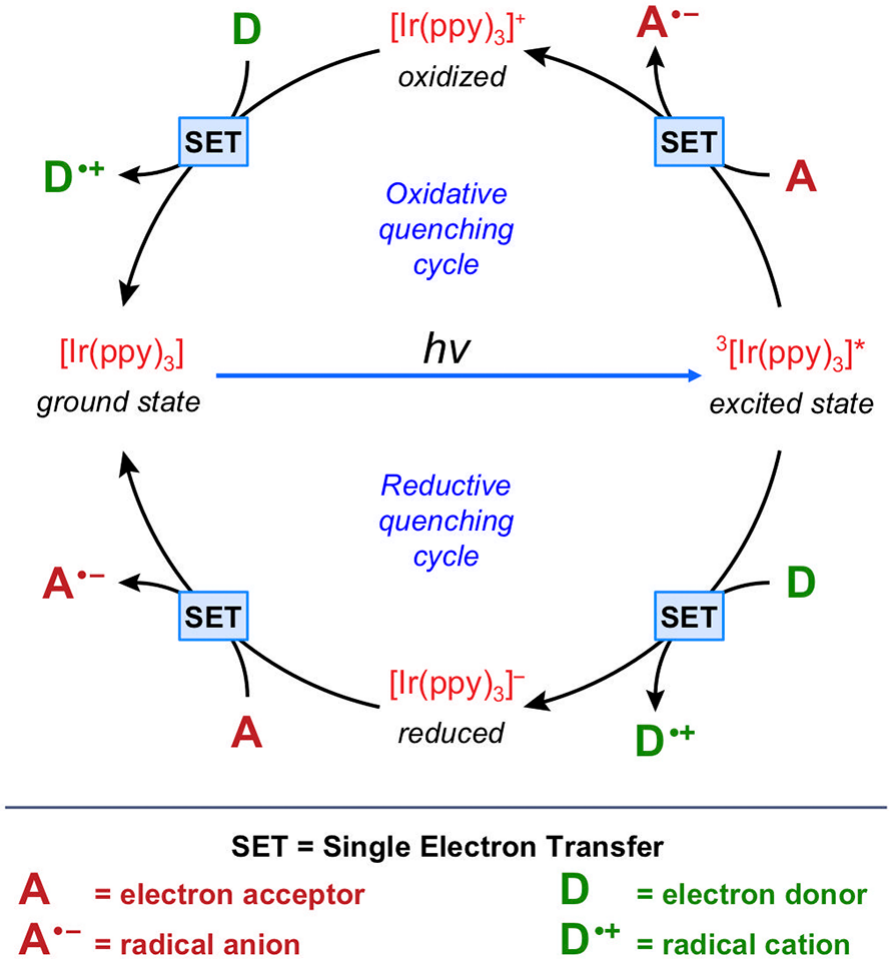
Oxidative and reductive quenching cycles for an archetypal Ir(III) complex.

Many of the commonly used photocatalysts are polypyridyl complexes based on Ru(II)^47,48^ and Ir(III),^46^ which are generally poor oxidants and reductants in the ground state.^42^ However, upon light absorption in the visible spectral region (i.e., at wavelengths where small organic molecules typically do not absorb), they afford stable and long-lived photoexcited states, which are highly redox-reactive. The conversion from stable complexes to highly redox-active species upon irradiation make them powerful tools for advanced catalytic processes (Figure 11).

The key parameters to qualify the capability of a given complex to serve as a photoredox catalyst are the excited-state oxidation and reduction potentials (E*_ox_ and E*_red_). They can be estimated from the ground-state potentials (*E*_ox_ and *E*_red_) and the energy gap between the ground and excited states (*E*_00_, the mean photon energy of the emission spectra in eV) through a simplified version of the so-called Rehm–Weller equation:^49^
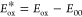
1
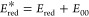
2Another important parameter is the excited-state lifetime, which must be long enough to warrant the effective encounter of the reactant with the catalyst, even at low (i.e., catalytic) concentrations.

In Table 1 are gathered the excited-state redox potentials of some of our above-discussed complexes, along with their emission maxima and lifetimes. They are compared with some of the most commonly used visible-light organic or organometallic photocatalysts. Despite the fact that eqs 1 and 2 provide only estimates of excited-state potentials (with uncertainties of 0.1 V or more),^50^ it is possible to make interesting comparisons. We have successfully tested [Ir(ptrz)_2_(dtbbpy)]^+^ (**33**) as an outer-sphere photoredox catalyst, allowing a highly selective 1,4-conjugate addition (Michael reaction) of radicals on a series of electrophilic olefins.^3^ Notably, in the case of the related fluorinated series (i.e., **30**, **31**, and [Ir(dfptrz)_2_(dtbbpy)]^+^ (**34**)),^4^E*_red_ is even more positive, making them the most powerful reductive photocatalysts among cyclometalated Ir(III) complexes and purely organic systems found in the literature, with the only exception being the Fukuzumi catalyst.^51^ Additionally, their excited-state lifetime is extremely long (i.e., 43.8 μs for **31**) compared to that of standard photocatalysts. This can reduce the amount of catalyst needed, trading off the limited light-harvesting capability of long-lived high-energy photocatalysts. Likewise, our anionic iridium complexes equipped with bis-tetrazole ancillary ligands (**19** and **20**) rank among the best photocatalysts in terms of E*_ox_.^2^

**Table 1:** Redox Potentials and Lifetime of Selected Visible-Light Photocatalysts

photocatalyst^a^	*E*_ox_ (V)^b^	*E*_red_ (V)^b^	*E*_00_ (eV)^c^	E^*^_ox_ (V)^d^	E^*^_red_ (V)^d^	λ_max_ (nm)^e^	τ (μs)^e^	refs
[Ru(bpy)_3_]^2+^	+0.88	–1.74	1.99	–1.11	+0.25	615	1.10	(42), (52)
*fac*-Ir(ppy)_3_	+0.36	–2.60	2.28	–1.92	+0.32	531	1.90	(53), (54)
[Ir(ppy)_2_(bpy)]^+^	+0.87	–1.78	2.00	–1.13	+0.22	602	0.28	(55)
[Ir(ppy)_2_(dtbbpy)]^+^	+0.91	–1.80	2.05	–1.14	+0.25	591	0.39	(55)
[Ir(fppy)_2_(dtbbpy)]^+^	+1.02	–1.84	2.19	–1.17	+0.35	552	1.06	(56)
[Ir(dfppy)_2_(dtbbpy)]^+^	+1.10	–1.87	2.32	–1.22	+0.45	519	1.35	(57)
[Ir(df(CF_3_)ppy)_2_(dtbbpy)]^+^	+1.31	–1.73	2.47	–1.16	+0.74	470	2.30	(58), (59)
[Acr^+^–Mes]	+1.65	–0.98	2.36	–0.71	+1.38	501	0.004	(51)
Eosin Y	+0.37	–1.47	2.19	–1.82	+0.72	556	0.004	(60−62)
PXX	+0.37	–2.56	2.62	–2.25	+0.06	447	0.005	(63)
4CzIPN	+1.11	–1.62	2.28	–1.17	+0.66	539	0.013	(64, 65)
NT	+1.34	–2.15	2.87	–1.53	+0.72	421	0.001	(66)
[Ir(ppy)_2_(b-trz)]^−^	+0.52	–2.64	2.37	–1.85	–0.27	498	2.09	(2)
[Ir(dfppy)_2_(b-trz)]^−^	+0.85	–2.51	2.50	–1.65	–0.01	467	2.27	(2)
[Ir(ptrz)_2_(bpy)]^+^	+1.16	–1.79	2.21	–1.05	+0.42	545	1.22	(37)
[Ir(ptrz)_2_(dtbbpy)]^+^	+1.14	–1.87	2.29	–1.15	+0.42	530	1.18	(37)
[Ir(ptrz)_2_(phen)]^+^	+1.18	–1.77	2.24	–1.06	+0.47	540	1.67	(37)
[Ir(dfptrz)_2_(bpy)]^+^	+1.45	–1.73	2.49	–1.04	+0.76	448	4.11	(4)
[Ir(dfptrz)_2_(dtbbpy)]^+^	+1.43	–1.82	2.50	–1.07	+0.68	448	4.90	(4)
[Ir(dfptrz)_2_(phen)]^+^	+1.45	–1.73	2.54	–1.09	+0.81	460	43.8	(4)

a dtbbpy = 4,4-di-*tert*-butyl-2,2-bipyridine; fppy = 2-(4-fluorophenyl)pyridinate; df(CF_3_)ppy = 2-(2,4-difluorophenyl)-5-trifluoromethylpyridinate; [Acr^+^-Mes] = 9-(2-mesityl)-10-methylacridinium perchlorate; Eosin Y = 2′,4′,5′,7′-tetrabromofluorescein; PXX = peri-xanthenoxanthene; 4CzIPN = 1,2,3,5-tetrakis(carbazol-9-yl)-4,6-dicyanobenzene; NT = 7-phenyl-6*H*-naphtho[2,3-*c*]chromen-6-one.

b Relative to Fc^+^/Fc, in acetonitrile (in some cases, adapted from the original works, according to *V*_Fc+/Fc_ = *V*_SCE_ – 0.41).

c Energy gap between the ground and excited states in acetonitrile, with an estimated error of ±0.1 eV.

d See eqs 1 and 2.

e Oxygen-free acetonitrile solutions, 298 K.

## 6 Conclusions

Heteroleptic complexes based on a third-row transition-metal ion such as Ir(III) combine a series of properties enabling a virtually unparalleled possibility to engineer the nature and energy of their relevant excited states. To this end, computational chemistry is a powerful tool for predicting electronic properties, inspiring molecular design, and more efficiently driving synthesis efforts. In this Account, we have illustrated our work in the area of heteroleptic cyclometalated iridium(III) complexes. This research was driven by several scopes: the elucidation of rational criteria for preparing robust luminescent materials via novel synthesis routes,^35−37,67^ blue-green emitters for light-emitting electrochemical cells,^2,37^ and efficient outer-sphere photoredox catalysts.^3,58^ Over the years, we gained a deeper understanding of the electronic and photophysical properties of these complexes, showing that they can be almost tailored by the design for a specific scope, typically in the areas of optoelectronics, sensing, and catalysis. We trust that this may inspire new work for further untapping the still vast potential of this unique class of photoactive metal complexes.

## Supporting Information Available

Photophysical data and details on the computational work to make the presented data uniform (PDF)
