# Supplementary material for: Excited-State Engineering in Heteroleptic Ionic Iridium(III) Complexes
Source: Acc Chem Res. 2021 Feb 22;54(6):1492–505. doi: 10.1021/acs.accounts.0c00825 (PMC9292135; doi:10.1021/acs.accounts.0c00825)
Supplement: Supplementary file 1 — ar0c00825_si_001.pdf [file ar0c00825_si_001.pdf]

Supporting Information

Excited-State Engineering in Heteroleptic Ionic Iridium(III) Complexes

Filippo Monti,[a] Andrea Baschieri,[a] Letizia Sambri,[b] and Nicola Armaroli[a,*]

[a] Istituto per la Sintesi Organica e la Fotoreattività, Consiglio Nazionale delle Ricerche, Via P. Gobetti 101, 40129 Bologna, Italy

[b] Dipartimento di Chimica Industriale "Toso Montanari", Università di Bologna, Viale Risorgimento 4, 40136 Bologna, Italy

*Correspondence to: nicola.armaroli@isof.cnr.it

Table S1. Photophysical and electrochemical properties of selected iridium(III) complexes

Complex	Emission data [a]			Electrochemistry [b]		Reference
	λ_{max} [nm]	τ [μs]	PLQY [%]	E_{ox} [V]	E_{red} [V]	
$[Ir(ppy)_2(bpy)]^+$ (**1**)	602	0.28	9.3	+0.87	−1.78	*Inorg. Chem.* **2011**, *50*, 11514-11526
$[Ir(ppy)_2(CN\textit{t}Bu)_2]^+$ (**5**)	453	32	52	+1.23	−2.38	*Inorg. Chem.* **2012**, *51*, 2263-2271
$[Ir(ppy)_2(CNtphNC)]^+$ (**12**)	452, 483	27.5	24	+1.23	−2.29	*Faraday Discuss.* **2015**, *185*, 233-248
$[Ir(ppy)_2(N\wedge C:)]^+$ (**14**)	474, 502	0.036	0.6	+0.86	−2.34	*Inorg. Chem.* **2013**, *52*, 10292-10305
$[Ir(ppy)_2(:C\wedge C:)]^+$ (**15**)	475, 503	2.28	37.5	+0.81	−2.54	*Inorg. Chem.* **2013**, *52*, 10292-10305
$[Ir(ppy)_2(trizpy)]^+$ (**16**)	574	0.059	0.5	+0.72	−2.02	*Inorg. Chem.* **2018**, *57*, 11673-11686
$[Ir(ppy)_2(triztriH)]^+$ (**17**)	490, 505	0.028	0.2	+0.72	−2.34	*Inorg. Chem.* **2018**, *57*, 11673-11686
$[Ir(ppy)_2(triztri)]^+$ (**18**)	498, 525	0.47	12.5	+0.44	−2.63	*Inorg. Chem.* **2018**, *57*, 11673-11686
$[Ir(ppy)_2(b\text{-}trz)]^-$ (**19**)	498, 520^{sh}	2.09	75	+0.52	−2.64	*Inorg. Chem.* **2017**, *56*, 10584-10595
trans-$[Ir(pimdz)_2(dmbpy)]^+$ (**24**)	606	0.28	8.5	+0.72	−1.95	*Inorg. Chem.* **2015**, *54*, 3031-3042
cis-$[Ir(pimdz)_2(dmbpy)]^+$ (**25**)	573	1.29	31.1	+0.82	−2.04	*Inorg. Chem.* **2015**, *54*, 3031-3042
$[Ir(trizpy)_2(b\text{-}trz)]^+$ (**26**)	470^{sh}, 499	3.77	11.7	+1.42	−1.82	*Inorg. Chem.* **2016**, *55*, 7912-7919
$[Ir(ptrz)_2(bpy)]^+$ (**27**)	545	1.22	54.8	+1.16	−1.79	*Inorg. Chem.* **2014**, *53*, 7709-7721
$[Ir(dfptrz)_2(bpy)]^+$ (**30**)	449, 480	4.11	75.9	+1.45	−1.73	*Inorg. Chem.* **2020**, *59*, 16238-16250

[a]The reported data have been obtained at room-temperature in acetonitrile, oxygen-free solutions. [b]Relative to Fc^+/Fc, in acetonitrile. sh=Shoulder

Computational details

All the density functional theory (DFT) calculations reported in this work have been repeated adopting the same theoretical model in order to allow rigorous comparisons between all the reported cyclometalated iridium(III) complexes, despite coming from different publications. All calculations were carried out using the B.01 revision of the Gaussian 16 program package[1] in combination with the M06 global-hybrid meta-GGA exchange-correlation functional.[2, 3] The fully relativistic Stuttgart/Cologne energy-consistent pseudopotential with multielectron fit was used to replace the first 60 inner-core electrons of the iridium metal center (*i.e.*, ECP60MDF) and was combined with the associated triple-ζ basis set (*i.e.*, cc-pVTZ-PP basis).[4] On the other hand, the Pople 6-31G(d,p) basis was adopted for all other atoms.[5]

All the reported complexes were fully optimized using a time-independent DFT approach, in their ground state (S_0) and lowest triplet states; all the optimization procedures were performed using the polarizable continuum model (PCM) to simulate acetonitrile solvation effects.[6-8] Frequency calculations were always used to confirm that every stationary point found by geometry optimizations was actually a minimum on the corresponding potential-energy surface (no imaginary frequencies). To investigate the nature of the lowest triplet states, geometry optimizations and frequency calculations were performed at the spin-unrestricted UM06 level of theory (imposing a spin multiplicity of 3), using the S_0 minimum-energy coordinates as initial geometries or other educated guesses (especially for finding ^{3}MC states).

All the pictures showing molecular geometries, orbitals and spin-density surfaces were created using GaussView 6 for Mac.[9]

1. Frisch, M. J.; Trucks, G. W.; Schlegel, H. B.; Scuseria, G. E.; Robb, M. A.; Cheeseman, J. R.; Scalmani, G.; Barone, V.; Petersson, G. A.; Nakatsuji, H.; Li, X.; Caricato, M.; Marenich, A. V.; Bloino, J.; Janesko, B. G.; Gomperts, R.; Mennucci, B.; Hratchian, H. P.; Ortiz, J. V.; Izmaylov, A. F.; Sonnenberg, J. L.; Williams; Ding, F.; Lipparini, F.; Egidi, F.; Goings, J.; Peng, B.; Petrone, A.; Henderson, T.; Ranasinghe, D.; Zakrzewski, V. G.; Gao, J.; Rega, N.; Zheng, G.; Liang, W.; Hada, M.; Ehara, M.; Toyota, K.; Fukuda, R.; Hasegawa, J.; Ishida, M.; Nakajima, T.; Honda, Y.; Kitao, O.; Nakai, H.; Vreven, T.; Throssell, K.; Montgomery Jr., J. A.; Peralta, J. E.; Ogliaro, F.; Bearpark, M. J.; Heyd, J. J.; Brothers, E. N.; Kudin, K. N.; Staroverov, V. N.; Keith, T. A.; Kobayashi, R.; Normand, J.; Raghavachari, K.; Rendell, A. P.; Burant, J. C.; Iyengar, S. S.; Tomasi, J.; Cossi, M.; Millam, J. M.; Klene, M.; Adamo, C.; Cammi, R.; Ochterski, J. W.; Martin, R. L.; Morokuma, K.; Farkas, O.; Foresman, J. B.; Fox, D. J. *Gaussian 16, Rev. B.01*, Gaussian Inc.: Wallingford, CT, USA, **2016**.
2. Zhao, Y.; Truhlar, D. G., The M06 Suite of Density Functionals for Main Group Thermochemistry, Thermochemical Kinetics, Noncovalent Interactions, Excited States, and Transition Elements: Two New Functionals and Systematic Testing of Four M06-Class Functionals and 12 Other Functionals. *Theor Chem Acc* **2008,** 120, (1-3), 215–241.
3. Zhao, Y.; Truhlar, D. G., Density Functionals with Broad Applicability in Chemistry. *Accounts Chem Res* **2008,** 41, (2), 157–167.
4. Figgen, D.; Peterson, K. A.; Dolg, M.; Stoll, H., Energy-Consistent Pseudopotentials and Correlation Consistent Basis Sets for the 5d Elements Hf-Pt. *J Chem Phys* **2009,** 130, (16), 164108.
5. Francl, M. M.; Pietro, W. J.; Hehre, W. J.; Binkley, J. S.; Gordon, M. S.; Defrees, D. J.; Pople, J. A., Self-Consistent Molecular-Orbital Methods .23. A Polarization-Type Basis Set for 2nd-Row Elements. *J Chem Phys* **1982,** 77, (7), 3654–3665.
6. Tomasi, J.; Persico, M., Molecular-Interactions in Solution - An Overview of Methods Based on Continuous Distributions of the Solvent. *Chem Rev* **1994,** 94, (7), 2027–2094.

7. Tomasi, J.; Mennucci, B.; Cammi, R., Quantum Mechanical Continuum Solvation Models. *Chem Rev* **2005,** 105, (8), 2999–3093.
8. Cramer, C. J.; Truhlar, D. G., Continuum Solvation Models. In *Solvent Effects and Chemical Reactivity*, Tapia, O.; Bertrán, J., Eds. Springer Netherlands: **2002**; Vol. 17, pp 1–80.
9. Dennington, R.; Keith, T. A.; Millam, J. M. *Gaussview, Version 6*, Semichem Inc.: Shawnee Mission, KS, USA, **2016**.
